# Argumentation: A calculus for Human-Centric AI

**DOI:** 10.3389/frai.2022.955579

**Published:** 2022-10-21

**Authors:** Emmanuelle Dietz, Antonis Kakas, Loizos Michael

**Affiliations:** ^1^Airbus Central R&T, Hamburg, Germany; ^2^Department of Computer Science, University of Cyprus, Nicosia, Cyprus; ^3^Open University of Cyprus, Latsia, Cyprus; ^4^CYENS Center of Excellence, Nicosia, Cyprus

**Keywords:** argumentation, position paper, human-centric approach, Artificial Intelligence, formal foundations, learning, reasoning, cognition

## Abstract

This paper aims to expose and analyze the potential foundational role of Argumentation for Human-Centric AI, and to present the main challenges for this foundational role to be realized in a way that will fit well with the wider requirements and challenges of Human-Centric AI. The central idea set forward is that by endowing machines with the ability to argue with forms of machine argumentation that are cognitively compatible with those of human argumentation, we will be able to support a naturally effective, enhancing and ethical human-machine cooperation and “social” integration.

## 1. Introduction

AI started as a synthesis of the study of human intelligence in Cognitive Science together with methods and theory from Computer Science.^1^ The general aim was to formulate computational models of human intelligence, and implement systems based on these models to emulate the natural form of intelligence. This original motivation was placed on the side lines in most of the middle years (1980–2010) of AI, with the emphasis shifting to super-intelligent AI (Bostrom, 2014) that could go beyond the ordinary human problem-solving capabilities within specific application domains, such as large-scale Planning (Bonet and Geffner, 2001), Data Analysis, and Data Mining (Nisbet et al., 2018).

The last decade has witnessed a return to the early AI goal of understanding and building human-like intelligent systems that operate in a cognitively-compatible and synergistic way with humans.^2^ This is largely driven by a growing market demand for AI systems that act as (expert) companions or peers of their human users. The reemergence of “old AI,” now called **Human-Centric AI (HCAI)**, aims to deliver services within the realm of natural or commonsense intelligence to support and enhance the users' natural capabilities in tasks ranging from organizing their daily routine, to ensuring compliance with legal or policy requirements, or to acquiring a first self-appreciation of a potentially troublesome medical condition.^3^

This ambitious vision for HCAI sets a challenging list of desiderata on the high-level characteristics that HCAI systems should exhibit. Table 1 gives an overview of a list of these characteristics.

**Table 1:** Major characteristics of HCAI systems.

**HCAI Characteristics**	**Description**
Human in the loop	At the level of design,
	development, and deployment of systems
Human-friendly behavior	Within the sphere of human-like
	modalities of interaction
Cognitive compatibility	At the different levels of its various
	groups of human users
Synergistic accountability	Explainable, contestable, and
	debatable operation and behavior
Embodiment of systems	In the physical, mental, and
	emotional human environment
Body-mind-like model	Of operation to sense,
	recognize, think, and act
Developmental nature	Of systems through a continuous process
	of learning and adapting from experience
Social integration	Transparently within the human society

But perhaps the most important desired characteristic of HCAI systems, overseeing all others, is: Adherence to human moral values promoting the responsible use of AI.

These vital characteristics for the development of HCAI systems attest to the need for a **multi-disciplinary** approach that would bring together elements from different areas, such as Linguistics, Cognitive Psychology, Social Science, and Philosophy of Ethics, and would integrate those into viable computational models and systems that realize a natural human-like continuous cycle of interacting with an open, dynamic, complex, and possibly “hostile” environment, and naturally enhance and improve their performance through their experience of operation and their evolving symbiotic relationship with their human users.

Building such HCAI systems necessitates a foundational shift in the problem-solving paradigm that moves away from the strictness and absolute guarantees of optimal solutions that are typically adopted for conventional computing, which are often brittle and break down completely when new information is acquired. Instead, HCAI would benefit by adopting **satisficing solutions** that strike an acceptable balance between a variety of criteria, are tolerant to uncertainty and the presence of incompatible alternatives, are robust across a wide range of problem cases, and are elastic in being gracefully adapted when they are found to have become inappropriate or erroneous in the face of new information.

This realization that intelligent solutions require the flexibility of accepting the possibility that errors can occur has been stated by Alan Turing, a forefather of Artificial Intelligence, at his lecture to the London Mathematical Society on 20th of February 1947 (Turing, 1947):

“[...] if a machine is expected to be infallible, it cannot also be intelligent.”

Accepting this realism of sub-optimal performance, HCAI systems would then use problem instances where they have experienced the fallibility of their current solutions to gradually adapt and improve the satisficing nature of those solutions.

The nature of HCAI systems under a new paradigm of accepting and tolerating reasonably-good solutions suggests new perspectives on the Learning and Reasoning processes, which operate together in synergy to produce intelligent behavior: a **new reasoning** perspective as a method of analyzing the acceptability of possible alternative solutions; a **new learning** perspective as a process of generating knowledge that can resolve the ambiguity in the data, rather than knowledge that draws definite predictions or defines concepts.

Although we have described these as new perspectives, they have essentially been present in AI for some time. The new reasoning perspective of not always arriving at conclusive or best conclusions is implicitly assumed by the areas of Non-Monotonic Reasoning and Belief Revision, proposed from the very start of AI, as essential elements of reasoning that would need to differ from formal classical reasoning. Similarly, the new learning perspective underlies, for example, the Probably Approximately Correct (PAC) Learning theory, where it is explicitly recognized that one can typically only approximate what one learns.

The inability of the new forms of learning and reasoning to reach a definitive answer is compensated in HCAI systems by the provision of **explanations** of the satisficing alternatives, which offer an account of the lack of (or inability to reach) best answers. This explanation-based interaction needs to be **cognitively compatible** with the human users and developers of the systems, in order to facilitate the integration of the various processes and entities that exist within the application environment.

**Figure 1:**
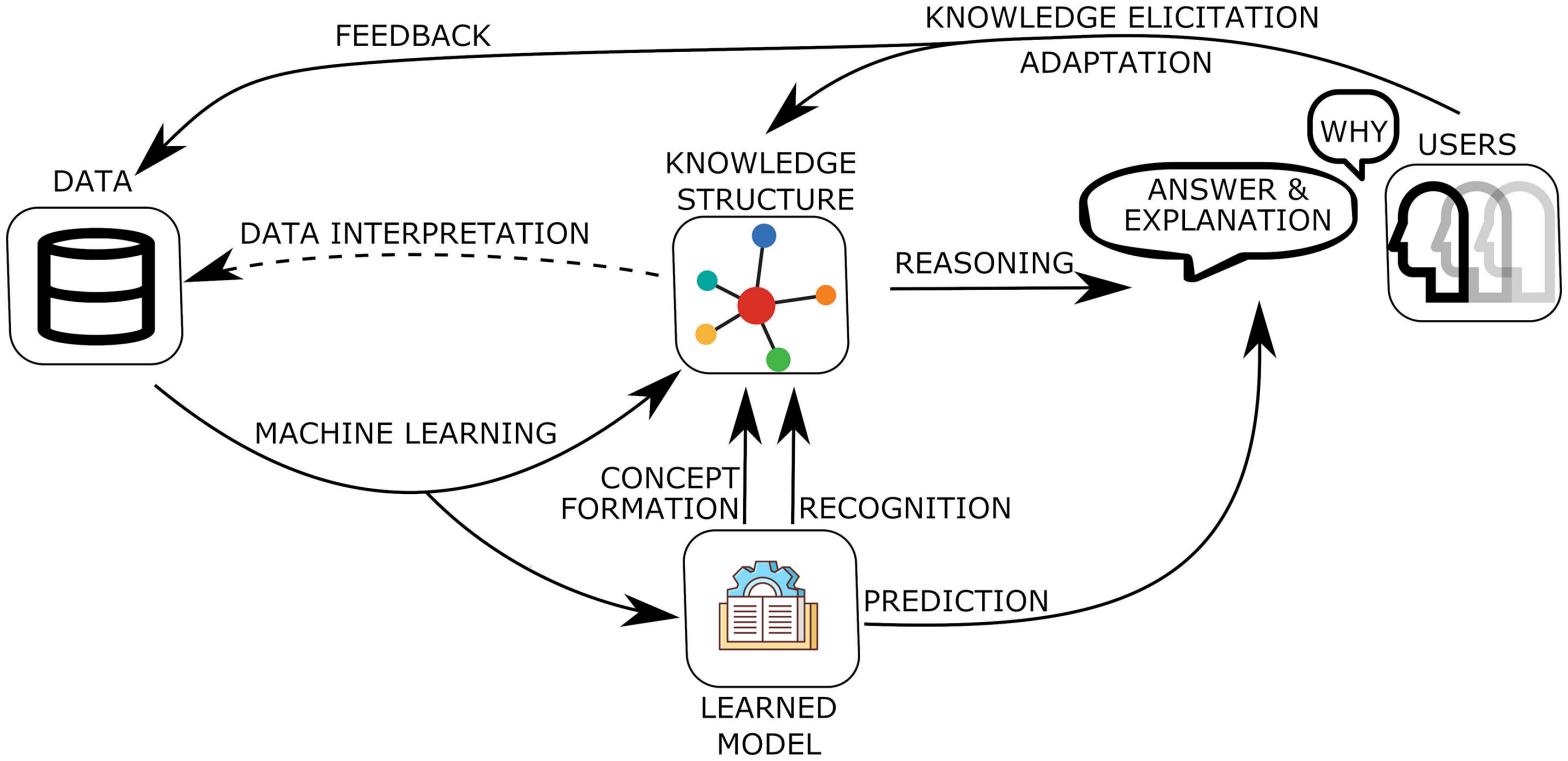
A high-level architecture of AI systems.

To help us place a human-centric perspective in today's terrain of AI research let us consider a typical high-level architecture of AI systems as shown in Figure 1. In this, learning and reasoning are tightly interconnected and both have a central role within the architecture. Learning is a continuous process that occurs throughout the life and operation of the system. **Machine learning** is used, e.g., in Deep Neural Learning, to generate structures for direct prediction, typically lower-level akin to *system 1* (Kahneman, 2011) in human reasoning. This could be identifying or **recognizing** some property of the current state of the environment to be combined with the general knowledge of the system or indeed to output a predictive classification directly to the user. Machine learning is also used at the symbolic level to learn the structure of and populate the knowledge of the system that is to be used for higher-level, akin to *system 2*, cognitive reasoning by the system. Recently, there is a strong interest in the integration of sub-symbolic and symbolic learning so that through such methods we have an emergent **concept formation** process of identifying and forming high-level cognitive concepts on top of the sub-symbolic learned structures.

Together with learning we can also use methods of **knowledge elicitation** from experts and/or users of the system to build the knowledge of a system and the general structure that we want the knowledge to have for our system. This is particularly useful at the initial stages of the development of a system and helps us to steer the development along a general form that we desire. For example, knowledge elicitation can be used to provide the basic guidelines for moral and ethical behavior of the system, which could then be continuously refined and **adapted** during its operation from its experience of interaction with the outside environment of users and the society in which it operates.

The system's interaction with its environment, which includes its developers and users, goes beyond simply providing the answers of its reasoning or prediction. It engages into a dialogue based on **explanations** of the system's **answer** at a level compatible with the way the human **users** (to which the explanations are addressed) themselves reason about the task. In order to have such meaningful interfaces, the **knowledge structure** of many AI systems is often connected to some structured form of Natural Language, so that its processing by the system can be linked to the human interpretation of the associated natural language form.

The development of an AI system is continuous with the **feedback** from its environment providing information to either revise and **adapt** the current state of its knowledge or to generate new data for further learning. For this development to be smooth it needs to take place under the prism of the current knowledge of the system. Hence, the results of reasoning by the system need to be explainable in terms of the current knowledge so that useful and meaningful feedback can be given to the system by its environment. Similarly, new experiences, that would drive new possibilities of learning, can first be interpreted under the current knowledge of the system to form suitable new data for further learning so that the new knowledge produced can fit naturally within the existing knowledge structure to which the system is committed. The development of the central element of the knowledge of an AI system is thus a matter of smooth evolution rather than a sequence of disconnected learning and adaptation processes.

**Paper position:** What is then an appropriate foundation for building HCAI systems with this variety of behavior characteristics and design features; a foundation that would give unity to the field and allow it to draw elements from several disciplines in order to synthesize coherent solutions to the challenge posed by HCAI?

We propose that such a foundation needs to be at the level of a new underlying logical framework, in an analogous way that Classical Logic is the foundation or Calculus for Computer Science (Halpern et al., 2001). Resting on the thesis (or hypothesis) that this logical framework should be built on a solid understanding of human **cognitive reasoning**, and acknowledging the natural link of argumentation with human cognitive reasoning and human decision making at large, this paper proposes **Argumentation** as the foundation or Calculus for Human-Centric AI.

The **aim of this paper** and its suggestion for the foundational role of argumentation in Human-Centric AI is to help bring together the wide variety of work on argumentation — ranging from argumentation in Philosophy and Ethics to the pragmatics of argumentative discourse in human debates — in order to understand how to synthesize a viable and robust basis for the development and use of HCAI systems. Systems that would meet their cognitive and ethical requirements, and integrate symbiotically, as expert or peer companions, within the human society, by complementing and enhancing the natural intelligence of humans.

The rest of the paper is organized as follows. Section 2 presents the general features of argumentation in support of the position of the paper. Section 3 gives a brief overview of the main components of computational argumentation, formalization and pragmatics, and illustrates the role of argumentation in HCAI systems with two example systems. Section 4 analyzes the main challenges that would need to be faced by any logical foundation of HCAI, linking to these challenges the features of argumentation that would be relevant in addressing them. Finally, Section 5 concludes by briefly discussing the importance of an interdisciplinary approach to HCAI.

## 2. Why argumentation as a logical foundation?

We ground the proposal for argumentation as a suitable logical foundation of HCAI on two observations about argumentation and their connection with the historical development of Cognitive Science and Logic-based AI.

The first such observation is the strong cognitive support for argumentation and its link to different cases of human thinking. This stems from many studies in Cognitive Science and Psychology, and based on experiments and theories that have widely compared human informal reasoning with classical formal reasoning (Evans, 2010). The early motivation of these works was to examine how rational, i.e., how close to strict logic, human reasoning is, and to record its deviation from the valid formal logical reasoning. In recent years, the paradigm changed from such normative theories, of how humans “ought to reason,” to descriptive theories, of how humans “actually reason.” Despite significant differences between the observed informal reasoning and the strictly valid formal reasoning, most humans are convinced that their way of reasoning is correct. Diverging from valid formal reasoning is often necessary to make intelligent decisions in everyday life!

An analogous shift can also be observed in Economics, from assuming the human as being “homo economicus,” i.e., an idealized rational agent in Neoclassical Economics, to accepting the bounded rationality of humans in Behavioral Economics, where the interest is in understanding how and why humans make decisions (Kahneman and Tversky, 1979; Paglieri and Castelfranchi, 2010) rather than modeling optimal choices. Decisions taken by people at large have been observed to deviate from logically strict or rational reasoning, and rather follow a heavily biased form of reasoning. Given the limited memory resources and time constraints of humans, the use of “efficient reasoning shortcuts,” such as biases or heuristics, are not only reasonable but necessary.

There is now strong evidence in various studies from Cognitive Psychology, brought together in the work of Mercier and Sperber (2011), that humans arrive at conclusions and justify claims by using arguments. With repeated experimental studies, Mercier and Sperber came to the conclusion that humans engage in motivated thinking through argumentation in order to defend their positions. In other words, argumentation is the “means for human reasoning.” Within the dual-process theory of human reasoning (Kahneman, 2011), with a *system 1* fast and intuitive process and a *system 2* slow and reflective process, Mercier and Sperber argue that “all arguments must ultimately be grounded in intuitive judgments that given conclusions follow from given premises,” in contrast to the usual assumption that *system 2* is unbiased and rather normative.

While in Cognitive Psychology and Behavior Economics the link to argumentation is examined following the scientific method of observation and theory formation, within the Humanities and particularly in Philosophy, scholars have been equating human informal reasoning with argumentation for centuries now. The entry on Informal Logic in the Stanford Encyclopedia of Philosophy (https://plato.stanford.edu/entries/logic-informal/) states:

“Though contributions to informal logic include studies of specific kinds or aspects of reasoning, the overriding goal is a general account of argument which can be the basis of systems of informal logic that provide ways to evaluate arguments. Such systems may be applied to arguments as they occur in contexts of reflection, inquiry, social and political debate, the news media, blogs and editorials, the internet, advertising, corporate and institutional communication, social media, and interpersonal exchange. In the pursuit of its goals, informal logic addresses topics which include, to take only a few examples, the nature and definition of argument, criteria for argument evaluation, argumentation schemes, [...,] and the varying norms and rules that govern argumentative practices in different kinds of contexts.”

Clearly, from the point of view of Humanities and other disciplines, human informal reasoning is a matter of argumentation.

The second main observation in support of argumentation concerns its relation and comparison with Classical Logic. The alternative of retaining Classical Logic, which has served conventional computing well over the decades, as the logical foundation for HCAI fails to capture fully certain forms of human reasoning that are well-outside the realm of formal classical logic. From the very early days of AI, the goal to address this discrepancy resulted in the search for and development of new logics for AI, such as non-monotonic logics, probabilistic, or fuzzy logics. In particular, a plethora of **non-monotonic logics** (Reiter, 1980; Shoham, 1987; Marek and Truszczyński, 1991) were proposed as candidates for the logical foundations of commonsense reasoning, starting with the logic of Circumscription for formalizing the Situation Calculus, a system for commonsense reasoning about the effects of actions and the change they bring about (McCarthy, 1968). These new logics aimed to capture the non-monotonicity feature of human reasoning, recognizing that, in contrast to formal Classical Logic, inferences should be flexible to missing or ambiguous information, and tolerant to (apparently) contradictory information, and should be possibly abandoned in the face of new relevant information.

Nevertheless, these new logics remained bound to the same formal and strict underpinning of Classical Logic making it difficult to deliver on their promise of “AI systems with commonsense” and human-like natural intelligence. On the other hand, the study of argumentation in AI, which was grounded on work in Philosophy and Cognitive Science (Toumlin, 1958; Perelman and Olbrechts-Tyteca, 1969; Pollock, 1987), showed that it was possible to reformulate (and in some cases extend) most, if not all, such non-monotonic AI logical frameworks (Bondarenko et al., 1997). Furthermore, it was recently shown that, within this AI approach to **Computational Argumentation**, it is possible to reformulate even Classical Logic reasoning as a special boundary case of argumentation, hence presenting argumentation as a universal form of informal and formal reasoning (Kakas et al., 2018; Kakas, 2019). These results together with the many links that Computational Argumentation has formed, over the last decades, with studies of argumentation in several other disciplines (see e.g., the journal of Argument and Computation^4^), have given a maturity to the field of Argumentation that allows it to serve as a candidate for the logical foundations of Human-Centric AI.

## 3. Computational argumentation: An overview

In this section we present a brief overview of (Computational) Argumentation, highlighting its elements that are most relevant to its possible foundational role for Human-Centric AI systems. This overview is built by considering elements drawn from the large corpus of work on Argumentation in AI over the last few decades.^5^ It concentrates on the essential elements of argumentation as a general logical system of human cognitive reasoning (or thought), avoiding technical details that may vary over different approaches and that are not crucial for understanding the central link of argumentation and reasoning.

Argumentation is a process of debating the alternative positions that we can take on some matter, with the aim to justify or refute a certain standpoint (or claim) on the matter. It can take place socially within a group of entities, with each entity typically taking a different standpoint and arguing its case, or within a single entity that contemplates internally the various standpoints in order to decide on its own stance. The process is **dialectic**, where in the social context it is carried out *via* an **argumentative discourse** within Natural Language in a debate between the different entities, whereas in the individual case this is done within an introspective internal debate within the thinking entity.

The dialectic process of argumentation takes place by (i) starting with some argument(s) directly supporting the desired standpoint, then (ii) considering the various counter-arguments against the initial argument(s), and (iii) defending against these counter-arguments, typically with the help of other arguments as allies of the initial arguments. The process repeats by considering further counter-arguments against these new allied defending arguments. We therefore have an “argumentation arena,” where arguments attack and defend against each other in order to support their claims, and the aim is to form a **coalition (or case) of arguments** that collectively supports “well” a desired standpoint. In forming such a coalition, we may need to include arguments that do not refer directly to the primary matter in question, but refer to secondary matters that have come into play through the initial stages of the argumentation process.

This arena of argumentation can be captured by a formal **argumentation framework**, which in an abstract form is a triple 〈Args,Att,Def〉, where Args is a set of arguments, Att is an **attack (or counter-argument)** binary relation between arguments, and Def a **defense (or defeat)** binary relation between arguments. Typically, the defense relation Def is a subset of the attack relation Att capturing some notion of the relative strength between the attacking arguments. Hence when $\left(a_{1},a_{2}\right)\in Def$ the argument *a*_1_ is strong enough to defend against (or defeat) *a*_2_.

In practice, abstract frameworks are realized by structured argumentation frameworks (Kakas and Moraitis, 2003; Gracía and Simari, 2004; Prakken, 2010; Modgil and Prakken, 2013), expressed as triples of the form 〈As,C,≻〉, where As is a set of (parameterized) **argument schemes** (Walton, 1996), instances of which form the arguments, C is a **conflict relation** between argument schemes (and between their arguments), and ≻ is a **priority (or preference or strength) relation** between argument schemes (and between their arguments). A structured argumentation framework, 〈As,C,≻〉 forms a **knowledge representation** framework, where knowledge is represented in a structured form, and on which the dialectic argumentation process of attack and defense can be performed.

Argument schemes^6^ in As are parameterized named statements of association between different pieces of information. They can be represented in the simple form of As=(Premises⊳Position), associating the information in the Premises with the statement of the Position. Hence, given the information in the Premises we can construct an argument (or reason) supporting the Position (or Claim) based on the link from the Premises to the Position in the argument scheme. The attack relation between arguments is constructed directly from the conflict relation C, which normally stems from some expression of incompatibility, e.g., through negation, in the underlying language of discourse. The defense relation is built using the priority relation ≻, where, informally, an argument defends against another argument if and only if they are in conflict and the defending argument is not of lower priority than the argument it is defending against. Importantly, and in contrast to the conflict relation which is static, the priority relation is **context-sensitive**, and depends crucially on (how we perceive) the current state of the application environment.

In computational argumentation, we impose a **normative** condition on which argument coalitions are considered **acceptable** as a **valid case** of support for their corresponding standpoints. This normative condition of acceptability stems directly from the dialectic argumentation process to examine and produce cases of support. Informally, an **acceptable** argument coalition is one that can defend against all its counter-arguments while not containing an internal attack between (some of) the arguments within the coalition^7^. In other words, attacking (or counter) arguments should be defended against, but in doing so we cannot introduce an internal attack between the arguments of the coalition.

This normative condition of acceptability of arguments gives a logical structure to argumentation. In comparison with Classical Logic, the **Logic of Argumentation** replaces the underlying structure of a truth model with that of an acceptably valid case of arguments. Logical conclusions are drawn in terms of the valid cases of arguments that support a conclusion. When a valid case supporting a conclusion exists we say that this is a **plausible or possible conclusion**. If, in addition, there are no valid cases for any contrary conclusion, then we have a **definite conclusion**.

Clearly, definite conclusions are closer to logical conclusions of formal logical reasoning systems, like that of Classical Logic. When they exist, definite conclusions are based on clear winning arguments in the argumentation arena, which ensure the strict and absolute consequence of the conclusion. This, then, corresponds to the **strict rationality** form of formal logical reasoning. For example, in the context of a decision problem where we require from the logic to identify rational choices for our decision, these definite conclusions would correspond to optimal choices. The Logic of Argumentation allows, in addition, a softer form of **Dialectic Rationality**, where several, typically opposing, conclusions (e.g., decisions) are considered rational as they are **reasonably justified** by an argument case that is valid. We thus have a more general form of rationality where the absolute guarantees of classical strict rationality are replaced by the accountability of dialectic rationality *via* the provision of a **justification** for the conclusion or choice. These justifications contain, in a transparent and explicit way, the different arguments that would render a conclusion **reasonable**.

Dialectic rationality depends on the **relative** importance we place on the various requirements of the problem at hand and the relative “subjective” value we give to the relevant information. Thus, a decision can be accepted as rational when it is reasonable under some set of standards or requirements, including the subjective preferences or biases that we might have for a specific standpoint. Concerns about a specific choice and the beliefs that underlie this are addressed in the dialectic argumentation process that has produced the argument coalition supporting that choice. Importantly, if new concerns are raised, e.g., by the dynamic application environment, then these should be addressed, and if the argument coalition for the choice cannot be adapted to address these concerns, i.e., to defend against the counter-arguments they raise, then the rationality of the choice is lost and as a consequence the suitability of the solution is lost.

### 3.1. Pragmatic considerations of argumentation

The feature of the Logic of Argumentation to naturally provide a justification for its conclusions is very useful within the **social context** of application of systems, as the justification can be turned into, and presented as, an **explanation** for the conclusion. The issue of providing explanations for the results of AI systems is today considered to be a major requirement for any AI system, and forms the main subject matter of **Explainable AI**. Explanations of conclusions, or taken decisions, serve well their social role of interaction when they give the basic reasons of support (attributive), they explain why a conclusion is supported in contrast to other opposing conclusions (contrastive), and they provide information that guides on how to act following the conclusion (actionable) (Miller, 2019).

Argumentation is naturally linked to explanation the recent surveys of Čyras et al. (2021) and Vassiliades et al. (2021) as well as the proceedings of the recent, first, International Workshop on Argumentation for Explainable AI (ArgXAI)^8^ give a thorough exposition of this link and its potential significance in AI. The arguments justifying a decision can form the basis of an explanation to another party. The argumentative dialectic reasoning process and the acceptable coalition of arguments that it constructs can be unraveled to give an explanation. Such explanations extracted from an acceptable argument coalition have an **attributive** element coming from the initial arguments that support the conclusion, while the defending arguments against the counter-arguments will provide a **contrastive** element of the explanation. These arguments also point toward taking (further) actions to confirm or question their premises, particularly when these relate to subjective beliefs or hypotheses.

As described above, the theoretical notion of **computation** that stems from the Logic of Argumentation, is that of the (iterative) dialectic argumentation process of considering arguments for and against an initial conclusion and other subsidiary conclusions that help to defend the arguments supporting the initial conclusion. During this dialectic process we have (at least) three choices that can render the process computationally intensive and highly complex. These complexity points are: the choice of initial argument(s), the choice of counter-arguments, and finally the choice of the defending arguments. The consideration of the **pragmatics of argumentation** (van Eemeren and Grootendorst, 2004) thus becomes an important issue when argumentation is applied in the real world. This includes questions of how are arguments activated and brought to the foreground of the argumentative process, and similarly how is the relative strength of arguments affected by the changing state of the external environment in which the process takes place.

To address this issue of the pragmatics of argumentation, we can draw from the large body of work on **Human Argumentation**, which studies how humans argue and how this results in the effectiveness that we observe in human reasoning. This study starts from Aristotle in the books of *Topics*, where he attempts to systemize argumentation and give detailed prescriptions of good practices for the way one can argue for or against a position. Recently, over the past decades, several works have set out detailed methods for formulating and understanding human argumentation from various different perspectives: philosophical, linguistic, cognitive, and computational; see the work of van Eemeren et al. (2014) for a comprehensive review. These include studies of understanding the various types of argument schemes that humans use in their argumentative discourse (Toumlin, 1958; Walton, 1996; Walton et al., 2008), or how the process of human argumentation relates to human reasoning (Pollock, 1987), and how human argumentation discourse can be regulated by pragmatic considerations that can help lead to agreement or a resolution of different standpoints in a debate (van Eemeren and Grootendorst, 2004).

Cognitive principles can then be drawn from these studies and from the study of human reasoning more generally, to be used as “cognitive guidelines” within the formal computational frameworks of argumentation to give a form of **Cognitive Machine Argumentation** that would be cognitively compatible with the argumentation and reasoning of humans (Saldanha and Kakas, 2019; Dietz and Kakas, 2021). This can then support an effective human-machine interaction *via* compatible forms of argumentation between machine systems and their human users.

Human argumentation is typically carried out in a social setting, as an argumentative discourse in Natural Language. It is, therefore, important to be able to recognize and extract the argumentation structure from the natural language discourse (Hinton, 2019, 2021). This includes the ability to recognize which parts of text are indeed argumentative, to identify the quality of the arguments that are extracted from the text, and, more generally, to extract the argumentative structure of support and attack between arguments extracted from various parts of some piece of text under consideration.

**Argument mining** is an area of study of argumentation which has strong links both with computational argumentation and with the study of human argumentation. It aims to automate the process of extracting argumentative structure (Lippi and Torroni, 2016; Lawrence and Reed, 2019) from natural language. It combines elements from the various different studies of human argumentation with methods from computational linguistics in order to turn unstructured text into structured argument data. This is typically carried out using an ontology of concepts relevant to some specific area of (human) argumentative discourse that we are interested in. Then applying argument mining on corpora of textual information related to a particular problem domain forms an important method to populate a computational argumentation framework for a corresponding application domain of interest.

Having described the basic idea behind Computational Argumentation and certain important connections to relevant lines of work, let us now illustrate, through two examples of candidate AI systems, how the Logic of Argumentation connects with Human-Centric AI. How would the Logic of Argumentation provide the basis for formulating and solving a Human-Centric AI problem?

### 3.2. Everyday assistants: Cognitive consultation support

Let us first consider the class of **Cognitive Review Consultation Assistants**, and more specifically a **Restaurant Review Assistant**, whose main requirement is to help human users to take into account the online reviews available on the various options in some decision problem. For simplicity, we will concentrate on how the logic of argumentation can help us use the information in the reviews for one particular restaurant in order to form a personal opinion about this restaurant. The problem of the assistant is to evaluate, but not necessarily to decide, whether the restaurant in question is a **reasonable choice** or not for a personal user of the system. A solution is an informed explanation of why the restaurant is a reasonable choice or not for the user based on the information on the reviews. Furthermore, we are not interested in identifying if a restaurant is an optimal best choice for us to dine out but rather a satisficing choice.

How can we represent this problem of the Restaurant Review Assistant in terms of an argumentation framework 〈As,C,≻〉? The argument schemes or arguments for and against a restaurant can be built using as premises the different types of information that the reviews contain. We will consider a **simple form of argument schemes** where these consist of a named association between a set of premises and an atomic statement of the supported position. To start with, the overall score of the reviews provides the premise for the basic arguments for the deliberation of the assistant: if the overall score is above some (personal) high threshold this will form an argument in favor of the restaurant, and if it is below some (personal) low threshold this will form an argument against the restaurant:


$$\begin{array}{c} As_{1}=\left(HighScore⊳Favorable\right)\\ As_{2}=\left(LowScore⊳Non\text{\_}Favorable\right). \end{array}$$

*HighScore* means that the score is above the high threshold, and *LowScore* that it is below the low threshold. Furthermore, when the overall score is in between these thresholds then we can have another two basic arguments, one supporting the position *Favorable*, and the other supporting *Non*_*Favorable*:


$$\begin{array}{c} As_{3}=\left(MiddleScore⊳Favorable\right)\\ As_{4}=\left(MiddleScore⊳Non\text{\_}Favorable\right). \end{array}$$

To complete the representation of the problem, we include in the conflict relation the obvious conflict between arguments that support the incompatible positions *Favorable* and *Non*_*Favorable*, and we leave the priority relation between these four arguments empty. In fact, the mutual exclusivity of the premises between most of the pairs of arguments, except between $As_{3}$ and $As_{4}$, makes the need to consider possible relative priorities essentially unnecessary. For the pair of $As_{3}$ and $As_{4}$, it is natural not to assign a relative priority between them. Hence, all conflicting arguments attack and defend against each other.

In general, the reviews will refer to, and comment positively or negatively on, properties that we usually consider relevant in evaluating the suitability of a restaurant: “service,” “cost,” “quality or quantity of food,” “atmosphere,” etc. Each such review would thus generate arguments for and against the suitability of the restaurant according to argument schemes of the following general form:


$$\begin{array}{c} As_{+ve}\left(Review\left(Id\right)\right)=\left(Positive\left(Property\right)⊳Favorable\right)\\ As_{-ve}\left(Review\left(Id\right)\right)=\left(Negative\left(Property\right)⊳Non\text{\_}Favorable\right). \end{array}$$

The premises of the resulting arguments are the positive or negative opinions that a review expresses on some of these relevant properties.

In general, the priority relation between these arguments would be mostly affected by the personal preferences of the human user, as communicated to their customized personal assistant, possibly through Natural Language guidelines, such as: *I prefer to avoid expensive restaurants, but I like to eat quality food*. With this statement, the user has identified the properties of “cost” and “quality” of food to be of particular relevance and importance, giving corresponding priority to arguments that are built with premises referring to these properties. Hence, a review that considers the restaurant expensive will give an argument built from $As_{-ve}\left(Review\left(Id\right)\right)$ higher priority than (some of the) other arguments for the position *Favorable*. But, as the guideline indicates, this argument will not have higher priority than arguments built using the scheme $As_{+ve}\left(Review\left(Id\right)\right)$ from reviews that stress the high quality of the food.

Given the aforementioned arguments, the dialectic argumentative reasoning simulates a debate between the various reviews (or possibly only a subset of the reviews chosen according to some criteria) and their positive and negative comments. Regardless of whether the assistant reaches a definite conclusion or remains with a dilemma on being favorable or not toward a given restaurant, the assistant will be able to provide an explanation based on the supporting arguments and the dialectic debate that has resulted in the acceptability of the argument according to the wishes of the user. These explanations will be very useful in the process of the assistant gaining the trust from its human user.

Cognitive Review Consultation Assistants are quite focused on very specific topics of interest. At a more varied level, we may want to build HCAI systems of “Search Assistants” to help us in getting a reliably balanced understanding on a matter that we are interested in. Eventually, Search Assistants should extract the arguments for and against the matter that we are interested in, together with their relative priorities, presenting to us a balanced view of the dialectic debate between these arguments. Tools and techniques from argument mining are directly applicable on, and a natural fit for, this extraction task, as one seeks to understand the argumentative discourse expressed in Natural Language, be that in the statements made by the human user in communicating their search parameters and preferences, or in the text or reviews that are being searched. For example, in the Reviews Assistant case, argument mining can be used (Cocarascu and Toni, 2016) to extract from the text of the reviews the arguments they are expressing, as well as the relative strength between these arguments, in support of positive or negative statements on the various features that are relevant for the user who is consulting the system.

### 3.3. Expert companion: Medical diagnosis support

Let us now consider another example class of Human-Centric AI systems, that of **Medical Diagnosis Support Companions**. This class of problems differs from the previous example of Everyday Assistants in that these systems are based on expert knowledge, on which there is large, but not necessarily absolute, agreement by the expert scientific community. Furthermore, these systems are not personalized to individual users, but they can have different groups of intended users. Their general aim will then depend on their user group. For example, if the user group is that of junior doctors in some specialization who need to train and gain practical experience in their field, then, within the framework of Human-Centric AI, these systems can have the general overall aim to:

“*Support clinicians feel more confident in making decisions, helping to avoid over-diagnosis of common diseases and to ensure emergency cases are not missed out.”*

Medical diagnostic knowledge that associates diseases with their observable symptoms can be represented in terms of argument schemes of the general form:


As=(Symptoms⊳Disease).

Hence, based on the premise that the information in *Symptoms* holds, we can build an argument that supports a certain disease (as the cause of the symptoms). For different sets of symptoms we would then have argument schemes that would provide arguments that support different diseases. These associations are expertly known and are treated as arguments, which means that they are not understood as definitional associations that must necessarily follow from the symptoms. Rather, for the same set of symptoms we can have argument schemes supporting different diseases, rendering each one of these diseases as plausible or suspicious under the same set of premises.

To complete the representation of the problem knowledge within an argumentation framework 〈As,C,≻〉, we would need to specify, in addition to these argument schemes, the conflict and priority relations. The conflict relation would simply capture the information of which diseases do not typically occur together. The priorities of arguments can come by following the diagnostic process followed by doctors in their practice of evidence-based medicine: Argument schemes as above apply on initial symptoms, e.g., the presenting complaints by a patient. Then the doctors have contextual knowledge of further symptoms or other types of patient information that allows them to narrow down the set of suspected diseases. This can be captured within the argumentation framework in terms of giving relative priority between the different basic argument schemes, where the priority is conditional on some extra contextual information.

In fact, one way to capture this contextual priority is in terms of preference or priority argument schemes, which support the preference of a basic argument for one disease over another basic argument for another disease, of the form:


$$As_{\text{prefer}}=\left(Context⊳\left(As_{1}≻As_{2}\right)\right),$$

where $As_{1}$ and $As_{2}$ are argument schemes supporting different diseases based on the same or overlapping premise information of symptoms and patient record.

Typically, the dialectic argumentation process would start between basic arguments supporting the alternative possible diseases, but then this is **entangled** with other dialectic argumentative processes arguing for the priorities of those basic arguments, and thus their ability to attack and defend, and so on. Hence, depending on the extra contextual information that is received by, or actively sought from, the environment, and the preference arguments that are enabled as a result, some of the diseases which were acceptably supported at the basic (general) level will not be so any more, if they are attacked by arguments supporting other diseases but with no defense available as before. Therefore, the set of suspicious diseases will be reduced, and the overall result will be that the diagnosis is further focused by this extra contextual information.

Another type of knowledge that can focus the result of the diagnostic process is contra-indication information, which supports the exclusion of some specific diagnosis. Such contra-indication information is typically strong and overrides other contextual information that would render a specific disease as being suspicious. This can be captured within argumentation in a similar way as above, by argument schemes that give priority to arguments against a specific diagnosis.

It is natural to compare this argumentation-based approach to medical diagnosis support systems with that of medical expert systems (Buchanan and Shortliffe, 1984) that were popular in the early days of AI. The knowledge in those early systems had to be carefully crafted by the computer scientists in terms of strict logical rules. Those rules, like the argument schemes we have described above, linked the symptoms to diseases^9^. The difference, though, with the argumentation-based representation, is that expert systems try to represent the knowledge in terms of logical definitions of each disease, a task which is very difficult, if not impossible, exactly because of the contextual differences that such definitions must take into account. For example, as definitions those rules would need complete information, and would need to ensure that there is no internal conflict or inconsistency among them.

The argumentation-based representation, on the other hand, can be incrementally developed by modularly adding new expert knowledge or by taking into consideration the feedback. This more flexible approach to knowledge representation is linked to the different perspective of HCAI systems, away from the expert systems perspective of reproducing and perhaps replacing the human expert, and toward the perspective of keeping the “human in the loop,” where the systems aim to complement and strengthen the human expert's capabilities.

## 4. Major challenges for Human-Centric AI

We now continue to describe some of the major challenges for the underlying logical foundations of Human-Centric AI and comment on how argumentation, in its role as a candidate for these foundations, relates to these challenges. We focus on presenting challenges at the underlying theoretical level of Human-Centric AI that would provide the basis for the principled development of systems, while we acknowledge that many other, more particular, technological challenges, would also need to be addressed to achieve the goals of Human-Centric AI.

The challenges for Human-Centric AI are not new for AI, but they reappear in a new form adapted to the human-centric perspective of HCAI. Overall, the main challenge for HCAI, and for AI more generally, is to acquire an understanding of human intelligence that would guide us to form a solid and wide-ranging computational foundation for the field. In particular, we need to understand thoroughly **Human Cognition**, accepting that the process of cognition, and its embodiment in the environment, form the central elements of intelligence.

This understanding of human cognition includes the following three important aspects: (1) how cognitive knowledge is organized into concepts and associations between them at different levels, and how cognitive human reasoning occurs over this structured knowledge, (2) how cognitive knowledge is acquired and learned, and how the body of knowledge is improved or adapted through a gradual and continuous development process, and (3) how the internal integrated operation of cognition, from low-level perception to increasingly higher levels of cognition, is supported by an appropriate architecture, and how an individual's cognition is integrated with the external physical and social environment. Below we will analyze separately these main challenge areas and discuss the inter-connections between them.

### 4.1. Knowledge and inference

Human-Centric AI systems are knowledge intensive. As in the case of human cognition, they will need to operate on large and complex forms of knowledge. To achieve this we need a framework for representing and organizing knowledge in structures that would facilitate appropriate types of inference and decision making. From one point of view (the anthropomorphic design and operation of AI systems), the task is to match the main features of Human Cognitive Knowledge and Reasoning, including their **context-sensitive** nature and the **multi-layered knowledge structure** into concepts and associations between them at different **levels of abstraction**.

The need for these characteristics of knowledge and reasoning had been identified from the early stages of AI, with various knowledge structures being proposed to capture them. For example, the structure of frames (Minsky, 1981) aimed to capture the context sensitive nature of knowledge. Similarly, inheritance networks (Horty et al., 1990) were used to capture the different cognitive levels of knowledge and a form of contextual inference based on hierarchical generalizations. Another such structure, that of scripts (Schank and Abelson, 1975), aimed to capture the context-sensitive nature of commonsense reasoning with the knowledge of stereotypical sequences of events, and the change over time that these events bring about. This approach of defining explicitly cognitive knowledge structures was replaced, over several decades up to the start of the 21st century, to a large degree by the search for **non-monotonic logics**. The emphasis was shifted away from suitable explicit structures in knowledge and the cognitive nature of the process of inference to that of rich semantics for these logics that would capture the intended forms of human cognitive reasoning. Intelligent reasoning would follow from the correctness of choice of the rich logical formalism.

Essentially, all these approaches were concerned with the major problem of the necessary adaptation of inference over different possible contexts. This challenge, named the **qualification problem**, was concerned with the question of how to achieve context-sensitive inference without the need for a complete explicit representation of the knowledge in all different contexts, and how this is linked to the desired inferences in each one of these explicitly represented general and specialized contexts. To address this problem of knowledge and reasoning qualification in non-monotonic logics, we would typically include some form of modalities and/or some semantic prescription in a suitable higher-order logic, typically over classical logic. The practical problem of turning the logical reasoning into a human-like cognitive inference in an embodied environment was considered to be of secondary difficulty by most of these approaches with some notable exceptions, e.g., in that of McDermott (1990).

Our proposal of argumentation as the logical calculus for Human-Centric AI assumes that an appropriate cognitive structure of knowledge can be captured within structured argumentation frameworks. This structure is given by the priority relation amongst the individual argument schemes, which expresses in the first place a direct and local form of qualified knowledge. This then induces implicitly a global structure on the knowledge *via* the attack and defense relations of argumentation that emerge from the locally expressed strength and conflict relations. The dialectic argumentative reasoning over this structure gives the qualification of inference over the various different and complex contexts. Indeed, Computational Argumentation, with its new approach to logical inference, was able to offer a unified perspective on these central problems of context-sensitive and qualified inference, by reformulating (and in many cases extending) most, if not all, known logical frameworks of non-monotonic reasoning in AI (Bondarenko et al., 1997).

The challenge for argumentation is to build on this, and understand more concretely the **argumentative structure of cognitive knowledge**, and how to use it to match the **practical efficacy** of human cognitive reasoning. For example, how do we recognize the context in which we are currently in so that we can debate among alternatives that are available in this context? Similarly, how do we recognize that there is insufficient current information that would lead to a reasonable inference? For example, there might be too many different conclusions that are equally supported, and hence we seamlessly recognize that it is not worth examining the inference, and it is better to wait for further information. This is akin to what humans naturally do in understanding narratives, where we leave empty pieces in the picture or model of comprehension, waiting for the author to reveal further information.

Another challenge related to the cognitive structure of knowledge is the need for a natural link to **explanations** for the inferences drawn at different cognitive levels of abstraction. In the organization of knowledge we can distinguish concepts that typically need explanation and those which do not — a separation that is also context sensitive depending on the purpose of the explanation and on the audience receiving the explanation. For example, the recognition of an image as a case of some abstract concept, e.g., of Mild Cognitive Impairment, can be explained in terms of some lower level features of the image, e.g., small HIP volume, which normally do not require (or for which one does not normally ask for) explanation. Perhaps one could ask for an explanation of “small” and be given this by some numerical threshold, in which case the even lower level feature of being less than the threshold is unlikely to be further questioned for an explanation. In any case, explanations need to be cognitively compatible with the user or process to which they are addressed, i.e., expressed at the same level of understanding and within the same language of discourse.

Argumentation has a natural link to explanation. Premises of arguments directly provide an attributive element of an explanation, while the structure of the dialectic argumentative process can be used to form a contrastive part of the explanations, i.e., explain why some other inference or decision was not made. This link of argumentation to explanation and the general area of Explainable AI has recently attracted extensive attention by the computational argumentation community (Kakas and Michael, 2020; Čyras et al., 2021; Vassiliades et al., 2021). The challenge is how to turn argumentation into the language of explanation in a way that the explanations are provided at an appropriate cognitive level and are of **high quality** from the psychological and social point of view, e.g., they are naturally informative and non-intrusively persuasive (Miller, 2019). Argumentative explanations can help the receiving process or human to take subsequent rationally-informed decisions, based on transparent attributive reasons for the rationality of a choice, while at the same time not excluding the freedom of considering or deciding on other decisions that are alerted to by the contrastive elements of explanations.

The high-level medium of human cognition, as well as the intelligent communication and interaction between humans, is that of **Natural Language**. The above challenges on the Structure and Organization of Knowledge and Reasoning need also to be related and linked with Natural Language as the medium of Cognition and Intelligence. Computational Linguistics and comprehension semantics and processes that are context-sensitive, such as the distributed semantics of Natural Language, are important in this respect to guide the development of AI. At the foundational level, the challenge is to understand cognitive reasoning on the medium of Natural Language. How is the process of human inference grounded in Natural Language, as it is studied, for example, in Textual Entailment (Dagan et al., 2009)? Several argumentation-based approaches study this question by considering how argumentative knowledge (arguments and strength) are extracted or mined from natural language repositories (Lippi and Torroni, 2016; Lawrence and Reed, 2019), i.e., how argument schemes are formed out of text (Walton, 1996), or how we can recognize good quality arguments (Hinton, 2019, 2021) from their natural language expression. The foundational challenge for argumentation is to understand how, in practice, the process of dialectic argumentation relates to and can be realized in terms of a human-like argumentative discourse in Natural Language.

### 4.2. Developmental nature

The recognition of the central role that knowledge plays in Human-Centric AI systems comes with the challenge of how that knowledge comes about in the first place, and how it remains current and relevant across varying contexts, diverse users interacting with the systems, and shifting and dynamic circumstances in the environment within which the systems operate. And all these, while ensuring that the knowledge is in a suitably structured form to be human-centric. Depending on the eventual use of knowledge, different ways of acquiring that knowledge might be pertinent.

In terms of a first use of knowledge, Human-Centric AI systems need to have access to background knowledge, through which they reason to comprehend the current state of affairs, within which state they are asked to reach a decision. Such knowledge can be thought to be of a commonsensical nature, capturing regularities of the physical or social world. Trying to fit empirical observations into a learned structured theory would be akin to trying to cover a circle with a square. The language of learning needs to be flexible enough to accommodate for the fact that not all empirical observations can be perfectly explained by any given learned theory. As obvious as this might sound, the majority of modern machine learning approaches implicitly ignore this point, and rather proceed on the assumption that the learned theory is a total mapping from inputs to outputs. As a result, these learning approaches are forced to consider richer and richer representations for learned theories (e.g., in the form of deep neural networks with millions of learning parameters to tune) that can, in principle, fit perfectly the learned data, losing at the same time the structure that one would wish to have in the learned theories, and opting for optimal rather than satisficing accuracy in their predictions at the expense of sub-par rather than satisficing efficiency.

An argumentation-based learned model, on the other hand, explicitly acknowledges that the learned theory only partially captures, in the form of sufficient conditions, whatever structure might be revealed in the empirical observations, choosing to abstain from predictions when these sufficient conditions are not met (e.g., for the areas of the circle that our outside the square). This is taken a step further, with these sufficient conditions not being interpreted strictly, but being defeasible in the presence of evidence to the contrary effect. Additional arguments in the learned model can thus override and fine-tune the conditions of other arguments (e.g., by pruning the corners of the square that might fall outside the circle).

By acknowledging the unavoidable incompleteness of a learned theory, a further related challenge emerges: the ability of a partially-good theory to be gracefully extended to a better one, without having to undertake a “brain surgery” on the existing theory. This elaboration tolerance (McCarthy, 1968) property allows one to adopt a developmental approach to learning, spreading the computationally demanding process of learning across time, while ensuring that each current version of the theory remains useful, usable, and easily improvable. An argumentation-based learned model can meet these requirements, as it can be gracefully extended with additional arguments, whose inclusion in the learned model is handled by the semantics of argumentation, without the need to affect the pre-existing theory. In case the extended part of the learned model comes in conflict with the original part, argumentation records that as a dilemma, and gives the learning process additional time to resolve this dilemma, even guiding the learning process on where it should focus its attention to be most effective.

In terms of a second use of knowledge, Human-Centric AI systems need to have access to decision-making knowledge, through which they reason to reach a decision on how to act in the current state of affairs, after comprehending that state with the aid of background knowledge. Such knowledge can be thought to be domain- and user-specific, capturing the preferences of the users of the system. It is expected, then, that such knowledge can be acquired by interacting with the users themselves whose preferences one wishes to identify.

In such an interaction, the system needs to employ a learning process that acknowledges the nature of human preferences, and the mental limitations of humans when communicating their preferences. Preferences might be expressed in a hierarchical manner (e.g., stating a general preference of red wine over white wine), with more specific preferences overriding the general preferences in certain contexts (e.g., when eating fish). Any preferences communicated by humans should, therefore, be taken as applicable in the absence of other evidence, but need to support their flexible overriding in the presence of exceptional circumstances or specific contexts.

At the same time, the preferences expressed by a human undertaking the role of a coach for the learner (Michael, 2019) should support their juxtaposition against social norms, ethical principles, expert knowledge, and applicable laws. Irrespective of whether such norms, principles, and laws are learned or programmed into a Human-Centric AI system, it should be easy to integrate them with the user's preferences that are passively learned or more directly provided by the user to the learner.

Since humans communicate most often in natural language, either with the explicit aim of offering their knowledge to a specific individual, or as part of supporting their position against another in a dialectical setting (e.g., in a debate in an online forum), the process of knowledge acquisition should be able to account for natural language as a prevalent source of knowledge. Techniques from argument mining (Lippi and Torroni, 2016; Lawrence and Reed, 2019) can be used to extract arguments directly from human discourse expressed in natural language. This discourse could represent the dialogue that a human has with the machine, in the former's effort to communicate their preferences to the latter. Equally importantly, the discourse can be undertaken in a social context among multiple humans. Mining arguments from such a discourse could help identify arguments in support and against diverging opinions on a matter, commonly agreed upon norms or principles, and, at a more basic level, the concepts that are deemed relevant in determining the context within which a decision should be made.

Fairness should be supported by the learning process by allowing the acquired knowledge to identify possible gaps, which might lead to biased inferences, so that the learning process can be further guided to fill these gaps and resolve the biases, by seeking to identify diverse data points from which to learn, and ones that would get learning outside any filter-bubbles. Relatedly, transparency should be supported by the learning process by ensuring that learned knowledge is represented in a form and structure that is compatible with human cognition.

Argumentation can identify gaps in knowledge, and sources of potential biases, by acknowledging that individual data points can form very specific and strong arguments that defeat the general arguments based on highly-predictive features, by having arguments dispute other arguments that rely on socially or ethically inappropriate features, and by supporting dilemmas in case the evidence for and against a certain conclusion might not be fully statistically supported. In all cases, the arguments in favor and against a certain inference can be made explicit to users, so that they can deliberate, for example, on the merits of high-accuracy coming through some rules, vs. the dangers of introducing biases.

A last, by major, overarching challenge for the process of knowledge acquisition is its meaningful integration with the process of reasoning. Learned knowledge does not exist in a vacuum, and it cannot be decoupled from how it will be reasoned with. Rather, during the learning process one has to reason with learned knowledge, so that its effects can be taken into account for the learning of further knowledge (Michael, 2014, 2016). This challenge is aligned with the challenge of learning structured and hierarchical knowledge, and the incremental nature of learning this knowledge. Once the bottom layers of knowledge are learned, they need to be used to draw intermediate inferences, so that the top layers of the knowledge can be learned to map those drawn intermediate inferences to higher inferences.

Not all layers of knowledge need to be represented as connections between identifiable concepts. At the lowest levels of learned knowledge, where inputs come in the form of unstructured (subsymbolic) data, neural architectures can play a meaningful role. As one moves from mapping those low-level inputs into identifiable concepts, one can then employ a representation that is based on symbols, enhancing the neural architecture with symbolic or cognitive layers of knowledge on top (Artur S. d'Avila Garcez, 2014; Tsamoura et al., 2021). Argumentation can take on the role of the language in which these cognitive layers of knowledge can be represented, allowing the necessary flexibility in mapping neural inputs to higher order concepts.

The developmental nature of learning, important in the context of building HCAI systems, has been studied in works on never-ending learning (Mitchell et al., 2015), curriculum learning (Bengio et al., 2009) and continual learning (De Lange et al., 2022), among others. Such works attempt to address the challenge that most current ML approaches face due to their batch-mode learning. If new data becomes available, previously trained knowledge is lost and the training process needs to start from scratch again. This process seems inefficient and improvable, in particular when we consider how humans learn over time. Mitchell et al. (2015) illustrate their suggested never-ending learning paradigm with the case of the Nevel-Ending Language Learner (NELL). NELL has continuously learned from the Web to read, and invents new relational predicates that extend the ontology to infer new beliefs. Bengio et al. (2009) take a different approach, what they call curriculum learning, but yet, similarly their motivation is inspired by human learning. They suggest to formalize training strategies, which define training orders, to reach faster training in the online setting and guide the training toward better regions in the parameter space to improve the overall quality of learning for deep deterministic and stochastic neural networks. Continual learning is yet another concept, where (De Lange et al., 2022) suggest to focus on artificial neural networks that can gradually extend knowledge without catastrophic forgetting.

Adopting argumentation as the target language of learning fits well with such attempts to develop continual learning processes (e.g., Michael, 2016). First, the take of argumentation on not producing definite conclusions in all cases is an explicit acknowledgment that any learned knowledge is never complete, and that learning is a never-ending process. When new data arrives, this can lead to new arguments, which can be seamlessly integrated into existing knowledge learned from previously available data. If the new data statistically support arguments in conflict with those previously learned, the semantics of argumentation handles the conflict by producing dilemmas, without leading to the catastrophic forgetting of previously learned knowledge. In addition, these dilemmas can naturally direct, through a form of self-driven curriculum learning, the learning process to seek additional data to resolve those dilemmas.

### 4.3. Internal architecture

The previously described challenges of how knowledge is organized to facilitate context-sensitive inferences and at the same time is naturally acquired such that knowledge adapts across domains and time, raises the question of how this is achieved, or wired, into the human mind.

For the classification of human experience and information processing mechanisms, Newell (1990) established the four bands of cognition, consisting of the biological band, the cognitive band, the rational band and the social band. These are characterized by the timescales of twelve different orders of magnitude. As an example, the time span of processes in the cognitive band can occur in 100 ms, whereas the time span of processes within the rational band ranges from minutes to hours. Newell was probably right when stating that any theory which only covers one aspect of human behavior “flirts with trouble from the start” (Newell, 1990), and therefore he suggested the development of architectures of cognition as formal structures in which different cognitive processes can be simulated and interact as modules.

At a general level, such **Cognitive Architectures** need to provide (i) a specification of the structure of the brain, (ii) the function of the mind and (iii) how the structure explains the function (Anderson, 2007). They are required to unify different information processing structures within one system that simulates the processes organized as modular entities and that are coordinated within one environment thus simulating human cognition and eventually predict human behavior. Over the decades, many cognitive architectures such as ACT-R (Anderson, 2007) or SOAR (Laird, 2012) have been proposed, which have had a significant contribution on providing formal methodologies and have been applied to various levels of cognition by including both symbolic and subsymbolic components. baseline model, the ‘standard model of the mind’ (or ‘common model of cognition’), in order to ‘facilitate shared cumulative progress’ and align theories on the architectural level.

However, even after 50 years, Newell's criticism that the scientific community does not “seem in the experimental literature to put the results of all the experiments together” (Newell, 1973) still seems to hold. Interestingly, this missing convergence toward unified theories of cognition persists across and within the *bands of cognition* (Newell, 1990). Bridging the gap between Newell's bands of cognition still exists as a problem and the main challenge remains. How do we organize the internal processes of a system at different levels such that they can operate internally linking perception and high-level cognition, by facilitating their meaningful integration with other systems and the external human participating environment? This is a question not only on how theories are embedded across levels, but also on which ones are adequate theories at the individual levels, and, in particular, on how organizational models are generated from theories across task domains.

The intention of HCAI to take the human perspective into account from the beginning of the system's development, in order to support and enhance the human's way of working, requires that its systems are judged not in terms of their optimization according to current AI performance criteria, but rather in terms of a holistic evaluation in comparison with the human mind and behavior. Laird, Lebiere and Rosenboom (Laird et al., 2017) emphasize that for human-like minds, the overall focus needs to be on ‘the bounded rationality hypothesized to be central to human cognition (Simon, 1957; Anderson, 1990)’. Accordingly, as we have stated several times in this paper, HCAI systems need to provide solutions that are not necessarily optimal in the strict rational sense but cognitively plausible across different levels. One way to address the above requirements is to build HCAI systems that have an internal representation of the current state of the human mind (Theory of Mind). This representation reflects the human's awareness of their environment from which plausible behavior in the given context can be ascertained. The system can consider the human perspective and generate their plausible decisions, if it has the ability to simulate the human's mind functions and their interaction with the simulated environment. Yet, the main challenge remains: How to organize the internal processes of a system at different levels such that they can operate internally in a coherent way and facilitate their meaningful integration with other systems and the external human participating environment. What is an adequate internal representation, and at which levels does the system need to be implemented? How are these levels organized internally?

Can Cognitive Argumentation help to address these challenges? Cognitive Argumentation has its foundations in Computational Argumentation and thus, at some level, its process of building arguments and the dialectic process of reasoning can be described and understood symbolically. Yet, the actual processes of building, choosing, and deciding which arguments are plausible or winners can be heavily guided by biases or heuristics which stem from lower level, e.g., statistical, components. These components might account for lower levels of cognition such as situation awareness or associative memory. Their connection with higher-level processes, such as the relative strength relation between arguments, can thus provide a vehicle of integration between internal system processes (e.g., Dietz, 2022). Cognitive Argumentation might therefore be considered as a good candidate for the internal integration, within appropriate cognitive architectures, of the processes at different cognitive levels of HCAI systems.

### 4.4. Social integration

Argumentation in practice is often a social activity, carried out through a dialogue or debate among (groups of) different individuals. Similar to a multi-agent system, where independent entities are understood as agents (passive, active, or cognitive), in an argumentation environment agents can be (groups of) individuals holding to or against a certain position. Multi-agent systems in their traditional sense have been used to study the dynamics of complex systems (e.g., economic systems) and the influence of different interactive behaviors among agents. Usually, the optimal outcome is computed with respect to a rational agent's behavior, i.e., an agent who selects an action that is expected to maximize its performance measure. In the case of Human-Centric AI systems, operating in such an optimality-seeking mode is not realistic. Yet, the different systems or agents need to operate within the same environment, either in a cooperative or competitive mode, as the case may be. The important challenge for this joint and social operation is sustainability, in the sense that individual systems can continue to provide their separate services while the ecosystem in which they belong continues to support their individual roles.

How can the logical foundation of argumentation facilitate achieving this goal of social sustainability? Argumentation can be understood as a multi-agent system where each agent (or group of agents) is a representative for supporting a certain position. The overall system might contain various (groups of) agents holding to different, possibly conflicting, positions. As in multi-agent systems, such an argumentation environment can have a notion of cooperation and competition. Cooperation can be understood as agents holding to the same position, where their joint goal is to defend their position or to convince others about their position. Competition is the case where agents have opposing positions and try to defeat the other's arguments, while defending their own arguments. Interaction among these (groups of) individual systems occurs through the arguments that defend their own positions or defeat the positions of others. This then can reflect the overall system's dynamics, which might either converge toward one position or stabilize to various (strong) positions that conflict with each other.

Another view on argumentation as a multi-agent system, following the work of Mercier and Sperber (2009), is to cast one agent as a communicator and other agents as the audience. The exchange of information happens dynamically through the persuasiveness of the communicator and the *epistemic vigilance* of the audience. In some sense this is the original context of the study of argumentation going back all the way to Aristotle who stages the process of dialectic argumentation between a Questionnaire and an Answerer. The motivation is to understand how to regulate the process of communication, e.g., exposing unreasonable positions and harmful rhetoric. In today's explosion of media and *social networks* this is particularly important in helping to enhance the quality of dialogue and interaction on these platforms (Heras et al., 2013; Gurevych et al., 2017). Recently, the center of Argument Technology (https://arg-tech.org/) has released a video exposing the dangers of harmful rhetoric, arguing that argumentation technology can help address this problem, e.g., with systems that support “reason checking” of the premises and validity of a position promoted on the media and social cyberspace.

In all cases, the approach needs to be strongly guided by cognitive heuristics (e.g., ‘bias by authority’, or heuristics concerned with the ethical aspects). The overall major challenge then remains the same. How can HCAI systems be socially integrated within an application environment for dialogue and debates? How can argumentation and the argumentative structure of knowledge facilitate such an integration?

### 4.5. Ethical compliance

The ethical requirement of HCAI systems is of paramount and unique importance. Its importance is reflected by the unprecedented interest and proactive actions that organizations and governments are taking in order to safeguard against possible unethical effects that AI can have on people's lives.^10^

One such EU initiative is the publication of “Ethics Guidelines for Trustworthy AI”^11^, prepared by a “High-Level Expert Group on AI,” suggesting that AI systems should conform to seven different requirements in order to be ethical and trustworthy (see also Floridi, 2014; Russell, 2019). At the systemic operational level, one of these requirements is that of the “Transparency: Including traceability, explainability and communication” of the system. This requirement alludes to the importance of AI systems being able to enter into a dialogue and a debate with human users or other such systems, and for this to be meaningful the system should be able to explain and account for its decisions and position. This will ensure some level of ethical behavior as through these processes of dialogue, dispute, and debate we will be able to identify ethical weaknesses and take action to remedy or mitigate the problem. The challenge then for any logical foundation of AI is to facilitate these processes and allow in a modular and natural way the adaptation of the systems with the results of the debate, either at the level of its knowledge, or at the level of its internal operation.

Transparency and other such requirements provide an operational approach to the problem. They do not touch, though, on the underlying foundational difficulty of what is good ethical behavior and how we can endow AI systems with it. The inherent difficulty in achieving the, otherwise simply stated, challenge of “AI systems that adhere to human moral values” lies in the fact that even if we are clear about the moral values by which we generally want to regulate our systems, in many circumstances we might have different moral values that are in conflict with each other.

The problem is not new. It is as old as Philosophy, where it was recognized that within ethical reasoning we can often have **moral dilemmas** of being unable to decide clearly what is the correct ethical decision or action to take. Socrates from the very early days of Philosophy raises this concern of morally difficult and unclear decisions depending on the particular context at hand, and Aristotle aims to give prescriptions for ethical reasoning in his Practical Syllogisms. Recently, in the context of AI, the Moral Machine project (Bonnefon et al., 2016) draws from the *miners dilemma* in Philosophy, in an attempt to gather data on the moral values of people and the relative importance they place on them, albeit within a very specific “AI context” that is directly relevant to the increasing prevalence of autonomous cars.^12^ The project confirms that decisions in ethical reasoning are not always clear and that they can vary between different people.

From this theoretical point of view it appears that the essential difficulty in this challenge for ethical decisions is that of capturing the context-sensitive nature of the reasoning involved. This is, therefore, the same problem described in Sections 4.1 and 4.2, where we have considered the nature of reasoning and learning in Human-Centric AI systems.

The flexibility of the Logic of Argumentation is well suited for the ethical guidelines, which although strong, they cannot be absolute, as situations can arise with genuine moral dilemmas (Verheij, 2016).^13^

In general, as we consider the challenge of how to develop the ethical quality within our AI systems, it would be useful to be able to judge the current degree of achieving this, i.e., what we could call the current level of **ethicacy** of a system.^14^ The form that this ethicacy measure would have depends on the logical perspective that we adopt about the ethical requirements, e.g., whether these are normative directives or guidelines to follow based on some descriptive principles. The normative view would point toward “ethics by design,” whereas the descriptive view would point toward an “evolutionary process.” Adopting the more flexible descriptive perspective, as argumentation would allow — instead of appealing to either ethics experts to prescribe, or supervised learning techniques to induce, the ethical principles — can support also a process of gradual acquisition of these principles. This process would resemble how young children learn from their parents and social surroundings: by being coached in an online and developmental manner as a reaction to their ethical transgressions (Michael, 2019, 2020).

Such a process of “ethics coaching,” be it by the user being assisted by the system, or by ethics experts acting on behalf of some community, or indeed special Ethics Coaching AI systems, can react to contest the decision of the system and possibly help to resolve the dilemma under some specified conditions. Critical in this interaction is that it is the justifications being evaluated, and not only the inferred conclusion, and that the reaction comes in the form of ethical counterarguments that do not completely nullify the system's current ethical principles, but complement them in an elaboration tolerant manner. Hence the ethical dimension of a system can start with some, pre-populated by design (by ethics experts) broad generally-accepted, ethical principles to guarantee some minimally-viable version of the system. Then, every time the system is faced with an ethically-driven dilemma on its material choices, the ethics coaching process will help the system, through a coaching dialogue on the justification of the alternatives, develop higher levels of ethicacy.

Argumentation, as a logical foundation supporting an ethical behavior, would allow machines to make transparent the reasons in favor and against the options available, and make transparent the ways in which these reasons are further developed and refined over time. Exposing the reasoning in one's decisions would seem to be the primary desideratum for an ethical system, over and above what the actual decision might end up being. At the end of the day, different people (or a system and a user) might disagree on their ethical principles. At the very least, argumentation can help expose the fundamental premises on which interlocutors disagree, even if it cannot help them reconcile their divergent views.

In his inaugural lecture,^15^ Verheij proposes not to regulate AI by enforcing human control or by the prohibition of ‘killer robots’, but through the use of argumentation systems which provide us with good arguments. *You cannot force good ethical behavior, you can only hope that you can form such behavior through exposure to the arguments for the alternatives*.

### 4.6. Summary of HCAI challenges

We can summarize these challenges by regrouping them into three main groups of different type of “technical requirements” expected from the logical foundations of HCAI systems and connecting to each one of these the main feature of argumentation that is appropriate to meet these requirements. Table 2 shows these three groups of requirements: **Openness** to capture the open nature of operation and development of the systems, **Humanly** to give the systems a human-like compatible behavior, and **Ethicacy** to capture the need for these systems to be effectively regulated by human moral values. In the second column of this table, we have the main corresponding features of argumentation that can help in addressing these requirements: The **flexible and non-strict** nature of argumentative logical inference together with the **online** process of argumentation are directly relevant in addressing the needs of the first group. For the second group of the requirements we note that the inference of argumentation is naturally **human-like**: human cognition and reasoning is naturally carried out through argumentation. The **dialectic** process of argumentation occurs in a framework of inner contemplation or debate between alternative points of view. This together with the natural link of arguments as justifications or explanations for supporting a view against others, can form the basis on which to build the required processes in the third group of the ethicacy requirements.

**Table 2:** Summary of technical challenges of HCAI, expected to be supported by its logical foundations and appropriate general properties of argumentation.

**HCAI Technical Challenges**		**Argumentation** **Properties**
Openness	Context-sensitive inference	
	Online and adaptive inference	Flexibility of
	Continuous and adaptive learning	argumentation logic
	Tolerance of inference to incompleteness	
	and conflicting information	
Humanly	Cognitively compatible
	system-human interaction	Argumentation-based
	Personalization of inference	human cognition
	Responsiveness to users feedback	
	Socially-driven inference	
Ethicacy	Cognitive explainability and transparency
	Contestable dialogues and debates	Dialectic nature
	Corrective moral/ethical coaching	of argumentation
	Osmotic learning of ethical behavior	

## 5. Conclusions

We have proposed Argumentation as a candidate for the logical foundations of Human-Centric AI. This position is based on the close and natural link of argumentation with human cognition. Argumentation as a formal system of reasoning could provide the underlying framework for computational models of human-like intelligent faculties for AI systems. The overall idea is that by allowing machines to argue, and by bringing their form of argumentation close to human argumentation, we can facilitate a smooth machine-human interaction that offers an enhancement of people's general intelligent capabilities in a natural way that is ethical and humane.

Whatever logic we choose, and no matter how appropriate we judge it to be, as a logical foundation for HCAI, this can only be the first step toward developing HCAI systems. Intelligence, whether human or artificial, is not a matter of pure logic as we are reminded by Kant and McDermott in their works “Critique of Pure Reason” (McDermott, 1990; Kant, 1998). A logical foundation needs to enable and facilitate the use of extra-logical cognitive information (or cognitive principles), in order to turn the underlying reasoning and learning that are supported by the logic into cognitive processes. Logic is not applied in isolation, but needs to be “aware” of a cognitive operational framework that affects and regulates its application. This cognitive embodiment would require the synthesis of knowledge from a wide range of disciplines that study the different aspects of human thought in its full generality.

**Figure 2:**
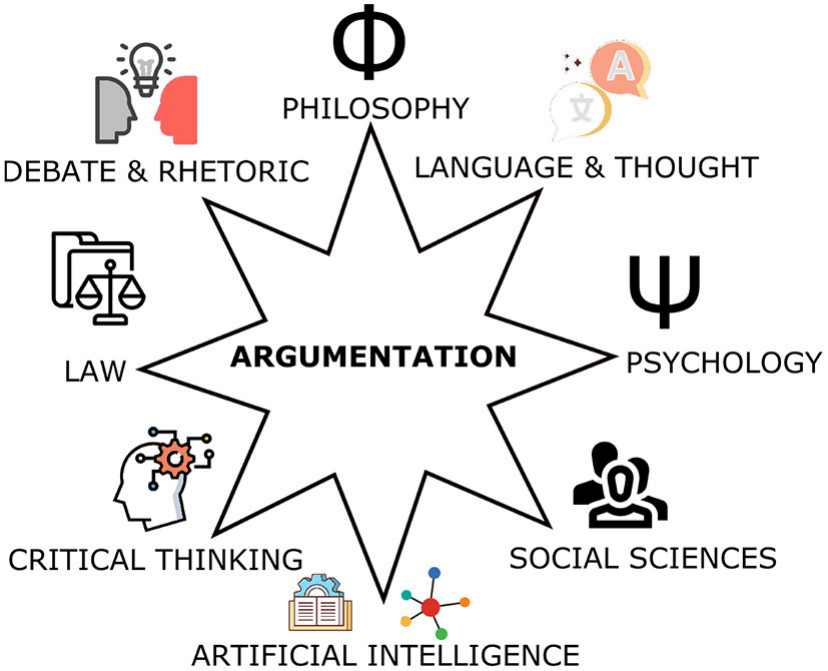
Overview of disciplines that study argumentation.

We are thus presented with an additional epistemological challenge, on top of the other technical challenges, of addressing the need for an interdisciplinary synthesis of the various studies of human argumentation under the perspective of Human-Centric AI. How can we draw from these different fields to form a foundation where machine argumentation is brought cognitively close to human argumentation? What empirical studies of human intelligence in these fields will help us understand its link with machine intelligence and particularly with computational argumentation, in a way useful for building HCAI systems? What elements of these fields are needed to allow the development of Human-Centric AI as a truly interdisciplinary field? For the case of argumentation, we are fortunate to have a wide ranging study of argumentation within several disciplines, such as Cognitive Psychology, Critical Thinking, Debate and Rhetoric, Argumentative Discourse in Natural Language, and studies of Practical Argumentation in different human contexts (see Figure 2). We can then draw from these studies to help us in addressing the interdisciplinary nature of HCAI.

Ideally, we would want this interdisciplinary synthesis to be so strong that Human-Centric AI would generate feedback into these other disciplines and become itself part of the general effort to understand human thought and intelligence. Can Human-Centric AI give a focus for pulling together the different efforts to comprehend human intelligence, and function as a new “laboratory space” for evaluating and further developing our understanding of the many different facets of human thought?

## Data availability statement

The original contributions presented in the study are included in the article/supplementary material, further inquiries can be directed to the corresponding authors.

## Author contributions

All authors listed have made a substantial, direct, and intellectual contribution to the work and approved it for publication.

## Funding

This work was supported by funding from the EU's Horizon 2020 Research and Innovation Programme under grant agreements (nos. 739578 and 823783) and from the Government of the Republic of Cyprus through the Deputy Ministry of Research, Innovation, and Digital Policy.

## Conflict of interest

The authors declare that the research was conducted in the absence of any commercial or financial relationships that could be construed as a potential conflict of interest.

## Publisher's note

All claims expressed in this article are solely those of the authors and do not necessarily represent those of their affiliated organizations, or those of the publisher, the editors and the reviewers. Any product that may be evaluated in this article, or claim that may be made by its manufacturer, is not guaranteed or endorsed by the publisher.

## Footnotes

^1^The Dartmouth workshop (http://raysolomonoff.com/dartmouth/), where the term Artificial Intelligence was introduced, was a joint meeting between scientists from the forming disciplines of Computer Science with Cognitive Science and other related areas.

^2^The recent book by Lieto (2021) describes the evolution of AI from the perspective of its link to human cognition, from its birth to today's developments.

^3^Today there are several centers dedicated to HCAI, such as https://www.humane-ai.eu/, https://hai.stanford.edu/, https://human-centered.ai/, https://humaine.info.

^4^ https://www.iospress.com/catalog/journals/argument-computation

^5^Work in the area of Computational Argumentation can be found in the journal of Argument and Computation and the International Conference on Computational Models of Argument (COMMA). Other sources for review material of the area include (Bench-Capon and Dunne, 2007; Simari and Rahwan, 2009; Atkinson et al., 2017; Vassiliades et al., 2021).

^6^Argument schemes are central to argumentation. They have been extensively studied, starting with Aristotle in his books of Topics, in various contexts of human argumentation. In recent times they are several important works that aim to standardize their form (Toumlin, 1958; Walton, 1996; Walton et al., 2008). In the work of Wagemans (2018) a periodic table for classifying the different arguments used by people is proposed.

^7^More generally, an acceptable argument coalition is one that once adopted can render all its counter-arguments non-acceptable.

^8^ https://people.cs.umu.se/tkampik/argxai/2022.html

^9^Note that this non-causal direction of association between symptoms and disease is the natural one when the knowledge is used in the practice of medicine, where doctors carry out the diagnostic process. The causal direction of association from a disease to symptoms is the natural direction when we are studying the underlying medical scientific theory.

^10^The EU is continuously releasing documents of guidelines and regulatory or legal frameworks on AI Ethics, e.g., https://digital-strategy.ec.europa.eu/en/library/proposal-regulation-laying-down-harmonised-rules-artificial-intelligence.

^11^ https://www.aepd.es/sites/default/files/2019-12/ai-ethics-guidelines.pdf

^12^ https://www.moralmachine.net/

^13^Also consider https://www.ai.rug.nl/~verheij/publications/oratie/oratie_Bart_Verheij.pdf, https://www.argnet.org/ethics-of-arg.

^14^Ethicacy: the efficacy in achieving ethical behavior; a measure of the ability to operate ethically to a satisfactory or expected degree.

^15^ https://www.ai.rug.nl/~verheij/publications/oratie/oratie_Bart_Verheij.pdf
